# A retrospective clinical analysis of pediatric paragonimiasis in a Chinese children’s hospital from 2011 to 2019

**DOI:** 10.1038/s41598-021-81694-7

**Published:** 2021-01-21

**Authors:** Manning Qian, Fei Li, Yuhan Zhang, Zhongwei Qiao, Yingyan Shi, Jun Shen

**Affiliations:** 1 Children’s Hospital of Fudan University, Shanghai, 201102 China; 2 Shanghai Medical College of Fudan University, Shanghai, 200032 China; 3 Department of Infectious Disease, Children’s Hospital of Fudan University, 399 WanYuan Road, Shanghai, 201102 China; 4 Department of Radiology, Children’s Hospital of Fudan University, 399 WanYuan Road, Shanghai, 201102 China

**Keywords:** Diseases, Medical research, Pathogenesis, Risk factors, Signs and symptoms

## Abstract

Diagnosis of pediatric paragonimiasis is difficult because of its non-specific clinical manifestations. We retrospectively reviewed the records of pediatric paragonimiasis in Children’s Hospital of Fudan University from January 2011 to May 2019. The confirmed diagnosis of paragonimiasis was based on positive anti-parasite serological tests from the local Center for Disease Control (CDC). A total of 11 patients (mean age: 7.7 ± 3.1, male–female ratio: 7:4) diagnosed as paragonimiasis were included. 81.8% were from endemic areas such as Sichuan and Yunnan, and 36% had a clear history of raw crab or crayfish consumption. The characteristic clinical features of pediatric paragonimiasis were eosinophilia (100%), pleural effusion (81.8%), hepatomegaly (54.5%), ascites (54.5%), and subcutaneous nodules (45.5%). Misdiagnosed with other diseases including tuberculosis (18.2%), pneumonia (9.1%), intracranial space-occupying lesions (9.1%) and brain abcess (9.1%) led to rehospitalization and prolonged hospitalization. For treatment, a 3-day course of 150 mg/kg praziquantel (PZQ) didn’t show ideal treatment effectivity and 63.6% needed more than one course of PZQ, while triclabendazole in a total dose of 10 mg/kg had a better efficacy to stubborn manifestations. This study indicated that pediatric paragonimiasis was often misdiagnosed, and the treatment with a 3-day course of 150 mg/kg PZQ had a high rate of failure.

## Introduction

Paragonimiasis is a food-borne parasitic infestation caused by the lung flukes of the genus *Paragonimus* spp.^1^. Infestation mainly occurs through the consumption of improperly cooked crab, crayfish, or raw meat of wild boar or deer infected with *Paragonimus* metacercariae^2^. According to the World Health Organization in 2015, the global burden of *Paragonimus* spp. was about 1.0 million disability-adjusted life years (DALYs)^3^. Human paragonimiasis has always been a serious medical problem in Africa, Asia and Latin America^4^. China, with a nationwide paragonimiasis prevalence rate of 1.7%^5^, has several epidemic areas such as Chongqing, Sichuan, Zhejiang, Yunnan and the region of the Three Gorges Reservoir^6^. Meanwhile, a large number of *Paragonimus* spp. was reported from China and the most predominant infections are *P.* westermani and *P.* skrjabini^7^*.*

According to the Chinese Center for Disease Control (CDC), information is limited in the symptoms, diagnosis and treatment for pediatric paragonimiasis. Diagnosis is often difficult in the cases of paragonimiasis for its vague and non-specific symptoms, especially in children who cannot accurately describe their symptoms and dietary history. It is easily confused with tuberculosis, fungal infection, malignant diseases, purulent meningitis and brain tumors^8–12^. However, misdiagnosis can lead to unnecessary medical treatments and procedures that can cause serious illness. Thus, our objective was to summarize the clinical manifestations of pediatric paragonimiasis, which are related to early diagnosis and treatment. Moreover, praziquantel (PZQ) and triclabendazole are two WHO-recommended drugs for the treatment of paragonimiasis for their curative effect and little adverse reaction. However, the Chinese CDC hasn’t differentiated doses and courses of treatment between children and adults^13^. So we try to find a suitable treatment regimen towards different severity and courses of pediatric paragonimiasis.

We retrospectively reviewed 11 cases of pediatric paragonimiasis who were diagnosed and treated at the Department of Infectious Diseases, Children's Hospital of Fudan University. Clinical information, treatment regimens and follow-up outcomes were collected. We aim to raise the awareness and understanding of clinicians towards this rare and neglected disease and give a discussion about the treatment of pediatric paragonimiasis.

## Results

### Patient demographics and clinical manifestations

Patient demographics and clinical manifestations for 11 patients were summarized in Tables 1 and 2. Among 11 patients included during a period of 8 years, 7 patients were males and 4 patients were females, with an average age of 7.7 ± 3.1 years (range, 3–12 years). The majority of them came from mountainous areas including Sichuan (54.5%), Yunnan (27.3%) and Zhejiang (9.1%), while 1 patient lived in the seaside of Zhejiang province. 45.5% presented with a history of consuming raw or undercooked crabs or crayfish or drinking unboiled stream water, 2 patients had swum in a river and 1 patient liked eating seafood, while 27.2% had no clear history associated with the disease.

**Table 1:** Characteristics of 11 patients infected with Paragonimus, China, January 2011–May 2019.

Patient no	Age, y	Sex	Location	Epidemiological information	Accumulative hospital stay, d	Misdiagnosis
1	3	F	Sichuan, mountainous area	Swimming in a river	8	NA
2	11	F	Zhejiang, seaside	In contact with raw seafood	9	Pulmonary tuberculosis
3	7	F	Sichuan, mountainous area	NA	11	NA
4	10	M	Yunnan, mountainous area	In contact with raw crabs	5	NA
5	7	F	Sichuan, mountainous area	In contact with raw crabs	7	NA
6	7	M	Sichuan, mountainous area	NA	15	Eosinophilic pneumonia
7	4	M	Yunnan, mountainous area	NA	33	Intracranial space-occupying lesion
8	10	M	ZheJiang, mountainous area	Favor of eating seafood	21	Tuberculosis
9	4	M	Yunnan, mountainous area	Swimming in a river	2	NA
10	10	M	Sichuan	History of raw crabs consumption	5	NA
11	12	M	Sichuan, mountainous area	History of mountain spring water consumption	50	Brain abscess

**Table 2:** The clinical manifestations of patients.

Characteristic	Value
**Signs and symptoms**	
Fever	5 (45.5)
Cough	4 (36.4)
Abdominal pain	3 (27.3)
Headache	2 (18.2)
Vomiting	1 (9.1)
Nausea	1 (9.1)
Localized weakness	1 (9.1)
**Physical examination**	
Subcutaneous nodules	5 (45.5)
Hepatomegaly	5 (45.5)
Lymphadenectasis	1 (9.1)
**Imaging findings**	
Pleural effusion	9 (81.8)
Ascites	6 (54.5)
Hepatomegaly	6 (54.5)
Hepatic nodules	3 (27.3)
Lymphadenopathy	3 (27.3)
Pericardial effusion	2 (18.2)
Pulmonary exudation	2 (18.2)
Splenomegaly	2 (18.2)
Abdominal wall mass	1 (9.1)
Brain edema	1 (9.1)
Cerebral calcification	1 (9.1)
Cerebral abscess	1 (9.1)
Cerebral nodules	1 (9.1)
Cerebral hemorrhage	1 (9.1)
Pulmonary cavity	1 (9.1)
Pulmonary consolidation	1 (9.1)
Pulmonary nodules	1 (9.1)
Penial nodules	1 (9.1)
Values are no. (%)	

In this group, 45.5% (5/11) were misdiagnosed as other diseases at other hospitals, including tuberculosis (18.2%, 2/11), eosinophilic pneumonia (9.1%, 1/11), brain abscess (9.1%, 1/11) and intracranial space-occupying lesions (9.1%, 1/11). Besides, the median hospital stay of patients misdiagnosed was 21 days (range 9–50), much longer than that of patients correctly diagnosed initially (7, 2–11). Before the diagnosis of paragonimiasis, patients received multiple unnecessary medications and treatments, and these were sometimes associated with serious illness.

Symptoms of the patients were summarized in Table 2. The common reasons for patients seeking hospital treatment were fever (45.5%, 5/11), cough (36.4%, 4/11) and abdominal pain (27.3%, 3/11), while neurological symptoms such as vomiting, headache, nausea, or localized myasthenia were relatively rare. Subcutaneous nodules (45.5%, 5/11) and hepatomegaly (45.5%, 5/11) were easy to find during physical examination.

### Laboratory findings

Blood routine examinations (RBTs) were performed in all patients. 90.9% (10/11) were detected with elevated peripheral blood WBC with an average of 13.90 × 10^3^/mm^3^ (range, 5.64–26.20) and the elevation of peripheral blood eosinophilia levels was present in 11 patients (100%) with the mean percentages of 38.25% (range, 5.80–74.00%) at admission. Anti-parasite serological tests were performed in 9 patients, and all of them showed positive serologic results for *Paragonimus* infection.

### Imaging findings

**Figure 1:**
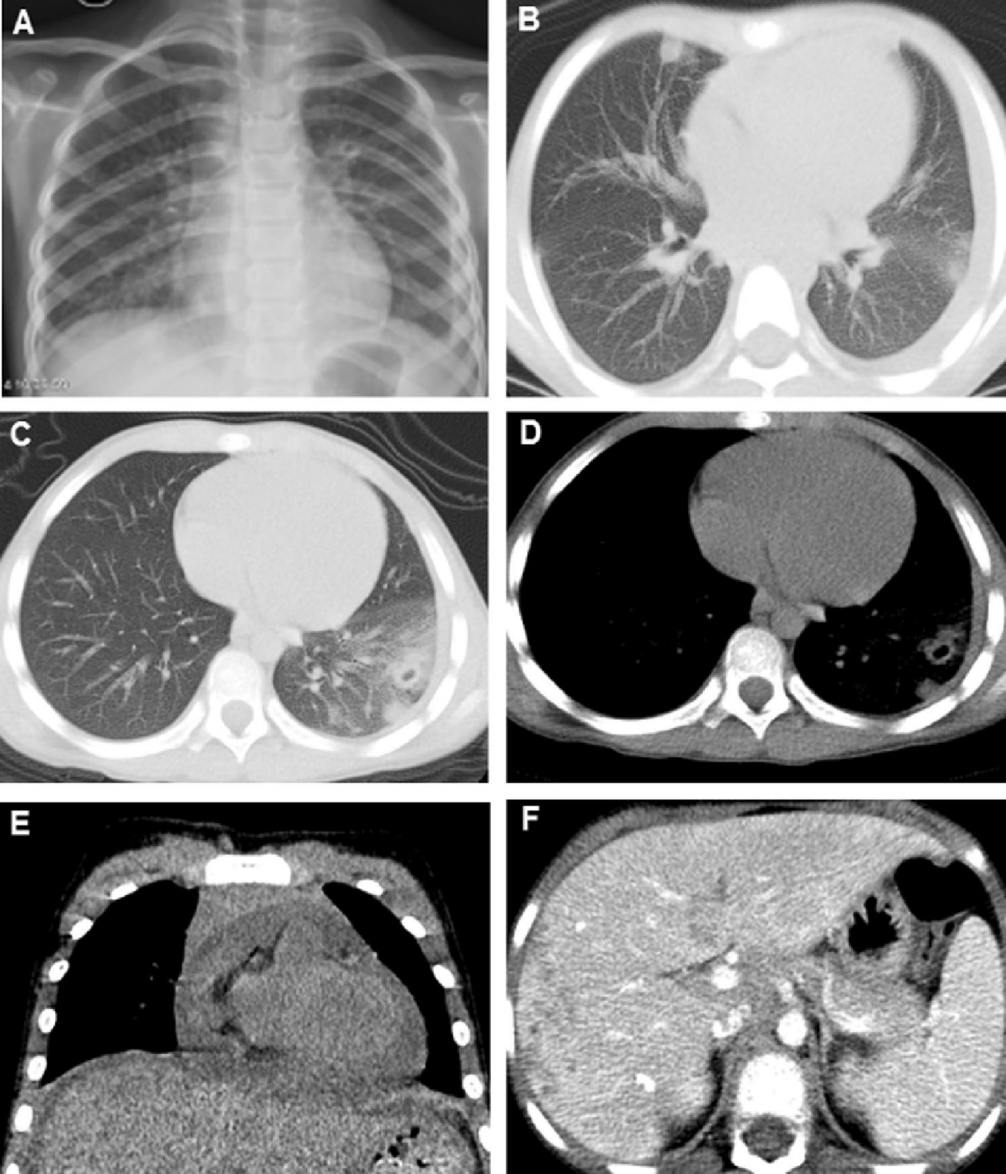
A 7-year-old girl presented with abdominal pain and subcutaneous nodules. (**A**) Chest X-ray showed pulmonary exudation in the right lower lung lobe; (**B**–**E**) Chest CT showed the alveolar opacification and ill-defined nodules in bilateral lungs, pleural effusion in both thoracic cavities; (**F**) Abdominal CT showed the multiple lesions with low density or calcifications in the liver.

**Figure 2:**
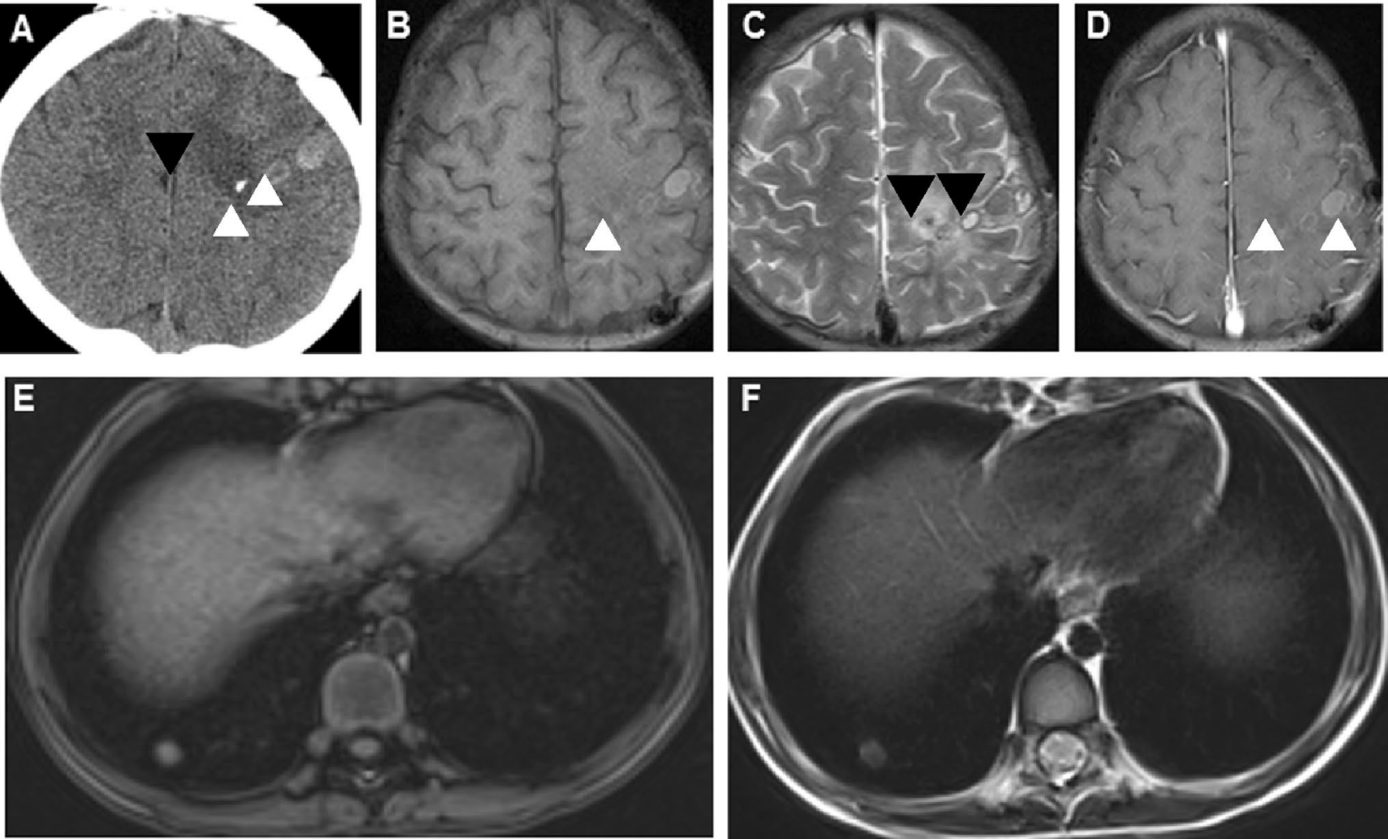
A 4-year-old boy presented with paroxysmal headache and vomiting. (**A**–**D**) Characteristic ring-like lesions with surrounding edema (white arrowhead) and calcifications (black arrowhead) located in the left frontoparietal lobe showed by cranial CT (**A**), T1WI (**B**), T2WI (**C**) and enhanced MRI (**D**); (**E**–**F**) Chest MRI indicated the nodules in the lower lobe of the right lung.

All the patients received imaging examination, abdominal ultrasonography for 8 patients, including cerebral CT for 5 patients, chest CT for 5 patients, chest X-rays for 4 patients, abdominal CT for 1 patient and echocardiogram (ECHO) for 4 patients. As a result, chest and abdominal CT had a higher detection rate, while ECHO had a lower detection rate (Table 3). Chest X-rays, coupled with chest CT have high sensitivity and accuracy for the diagnosis of paragonimiasis. Among 4 patients accepted chest X-rays, pleural effusion was the most common finding. For patients without abnormalities on X-ray plain films, chest CT would be used for more detailed anatomical observations including pleural effusion, alveolar opacification and ill-defined nodules (Fig. 1A–E). For hepatic paragonimiasis, hepatomegaly and ascites were easily found through abdominal ultrasonography and multiple lesions with low density or calcifications in the liver were found through abdominal CT (Fig. 1F). Patients with cerebral paragonimiasis were diagnosed through cerebral CT and MRI (Fig. 2). Brain edema, cerebral calcifications, abscess, nodules and cerebral hemorrhage were common signs for cerebral paragonimiasis (Table 2). Detailed imaging findings are presented in Table 2.

**Table 3:** Common imaging scans and its positive detection rate.

Imageological examination	No. (%)	Positive rate (%)
Abdominal ultrasonography	8 (72.7)	86
Cerebral CT	5 (45.5)	40
Chest CT	5 (45.5)	100
Chest X-ray	4 (36.4)	75
Abdominal CT	1 (9.1)	100
ECHO	4 (36.3)	25

### Treatment and follow-up outcomes

All 11 patients were treated with at least a standard course of PZQ. However, 63.6% didn’t respond well with a single course of PZQ treatment and needed another course of 3-day PZQ therapy. All patients were required to return for outpatient follow-up, however, 3 of them were lost to follow-up. Hematological, serological or imageological improvements were detected in the follow-up period of most patients with several courses of praziquantel therapy. Nevertheless, a patient treated with three courses of PZQ in a total dose of 150 mg / kg, three times a day, given orally for 3 days still had subcutaneous and penial nodules and eosinophilia (Table 4). Then, the patient accepted a course of triclabendazole in a total dose of 10 mg/kg orally, and as a result, subcutaneous and penial nodules eliminated and eosinophils count returned to normal. 3 patients required rehospitalization and 2 of them were misdiagnosed with lower age, 3 years and 4 years respectively.

**Table 4:** Changes in peripheral blood of a patient with subcutaneous and penial nodules and eosinophilia after 3 courses of PZQ.

Date	EOS (*10^9^)	EOS(%)	WBC (*10^9^)	N (%)
2017.8.17	4180	32.4	12.9	27.7
2017.8.25	4020	30.9	13.0	29.7
2017.9.7	2910	21.6	13.5	38.6
2017.9.19	4910	26.1	18.8	39.1
2017.10.5	2450	19.0	12.9	34.2
2017.11.7	6020	29.8	20.2	42.8
2017.12.19	6580	39.6	16.6	29.5
2018.1.18	760	7.2	10.6	42.7
2018.2.22	1880	17.0	11.1	33.0
2018.5.3	3910	26.9	14.5	32.4
2018.5.3	Given a course of triclabendazole			
2018.8.16	1860	11.5	16.2	49.2
2019.2.11	4110	31.3	13.1	32.8
2019.5.23	1350	13.2	10.2	40.8
2019.7.11	2370	19.0	12.5	40.2

## Discussion

Data are limited in the incidence, diagnosis and treatment of children with paragonimiasis. After infection with *Paragonimus* spp., clinical symptoms usually appear after 2–20 days. When metacercariae migrate to different parts of the body, patients will present with fever, fatigue, diarrhea or upper abdominal pain and eosinophilia^14^. Immature forms migrate through the duodenal wall, peritoneal cavity, and diaphragm to become encapsulated and mature within the lungs, or other ectopic tissues including the liver, brain, kidneys, adrenal glands and peritoneal and mesenteric lymph nodes^2,5,7^. Therefore, symptoms of paragonimiasis were usually variable, as agents of a “neglected tropical disease.” Our study concluded the clinical and imaging findings of paragonimiasis and its treatment course to improve the awareness of this disease.

In this study, among all 11 cases of paragonimiasis in children, all of them came from epidemic areas including Sichuan, Zhejiang and Yunnan and none of them came from Shanghai, which was inconsistent with the report that big city had higher incidences of paragonimiasis^15^. Dietary habits of raw or undercooked food are also prevalent in these cities^16^. In our study, 45.5% of the patients presented with a history of consuming raw or undercooked crabs or crayfish, and 2 patients may be contacted with metacercariae-contaminated stream when swimming, while 27.2% of the patients had no clear history associated with the disease. Our findings are consistent with other two large reports of pediatric paragonimiasis in China^13,16^. Gong et al*.* reported that 58.5% (72/123) had a history of consuming freshwater crabs and Xu et al*.* reported that 79.3% (46/58) admitted a history of raw water or food ingestion. Changing dietary patterns are having a profound effect on the epidemiology of paragonimiasis^17,18^. Hence, it is necessary to appeal to change risky eating habits and strengthen the awareness of not taking raw water or food.

Diagnosis is difficult in paragonimiasis because of its non-specific and vague symptoms. Fever and subcutaneous nodules are two main complaints of patients at admission instead of gastrointestinal symptoms or respiratory symptoms reported by Gong et al*.*^13^. Fever is regarded as a sign of early infection of *Paragonimus,* which can appear in as little as 2–4 days after ingestion of metacercariae^19^. Subcutaneous nodules, especially migratory subcutaneous nodules, are an important manifestation of extra-pulmonary paragonimiasis, which is caused by the aberrant migration of juvenile worms. For imaging findings, pleural effusion as an important clinical manifestation of pulmonary paragonimiasis appeared in 81.8% of patients in our group, suggesting that most pediatric patients with extra-pulmonary paragonimiasis also had pulmonary paragonimiasis in our group. Moreover, patients with cerebral paragonimiasis showed characteristic ring-like lesions with surrounding edema in MRI and they often had neural symptoms including vomiting, dizziness or cephalalgia, which is different from Xu et al*.*’s results^16^. Also, eosinophilia is a very frequent finding in parasite infections. Many functions and properties have been attributed to eosinophils such as exocytic attacks on helminths via released substances, pro-inflammatory effects as well as mechanisms to modulate immune processes^20^. In our study, eosinophilia was noted in 11(100%) patients and elevated WBC count in 10(90.9%) patients, higher than data reported by Xu et al*.*^16^ and may related to the severity of the disease. The change in peripheral blood eosinophil was related to the course of the disease, as seen in Table 3, suggesting that the change in the eosinophil level may be used as a sign for the evaluation of anti-infection therapy.

Misdiagnosis is common, especially in children who cannot exactly describe dietary history and symptoms. In our study, the misdiagnosis rate at admission was 45.5%, and the most commonly confused disease was tuberculosis due to their similar symptoms and signs. Moreover, some clinical manifestation of extra-pulmonary paragonimiasis such as space-occupying lesions in the liver, cerebrum or spinal canal can be easily misdiagnosed with tumors^11,21,22^. Delayed diagnosis is associated with serious illness, as shown in our results via median hospital stay and rehospitalization. The reason for a prolonged hospital stay can be unnecessary medications and treatments patients received before the diagnosis of paragonimiasis. Therefore, there is a need for a better understanding of relatively rare lung paragonimiasis infection in pediatric patients. Paragonimiasis should be considered in patients with symptoms of intermittent fever, cough or subcutaneous nodule, especially in those come from epidemic regions, have a history of raw crabs or crayfish consumption, or present elevated eosinophil counts.

PZQ and triclabendazole are two WHO-recommended drugs for the treatment of human paragonimiasis. According to the recommendations of the American Academy of Pediatrics, PZQ is the first line of treatment for paragonimiasis and the recommended course is a total amount of 150 mg/kg given three times a day orally for three days^23^. In our study, a 3-day course was used to alleviate side effects such as allergic reactions and brain edema, brain herniation after high-dose therapy. However, the 3-day course didn’t show ideal treatment effectivity and 63.6% patients needed more than one course of PZQ, much higher than that reported by Gong et al. (23.6%)^13^. The high rate of misdiagnosis and severe symptoms of patients in our group may be also related to low response to PZQ. It has been reported that paragonimiasis associated with effusions have unsatisfactory responses to the initial PZQ treatment^24,25^, then 81.8% in our group had pleural effusions. One patient whose subcutaneous nodules still existed after 3 courses of PZQ treatment received a total dose of 10 mg/kg triclabendazole. Furthermore, triclabendazole treatment had a satisfactory therapeutic effect for eliminating subcutaneous nodules. We were the first medical institution in China who had reported using triclabendazole for a pediatric patient with paragonimiasis.

Our study had several limitations. First of all, our sample size was small and it was from one pediatric center, thus the results had extrapolation biases. Besides, the case history of some patients transferred to another hospital in the midway was not detailed. Future studies of pediatric paragonimiasis should be investigated over the country.

In conclusion, the clinical manifestations of paragonimiasis are lack specificity, thus commonly leading to misdiagnosis. Therefore, the diagnosis should be combined with epidemiological history, clinical manifestations and laboratory results. Treatment strategy should be refined to different severity and course of the disease and more investigation on the treatment regimen is needed to improve the curative. When a 3-day course of 150 mg/kg PZQ had a high rate of failure. Triclabendazole would be another choice.

## Methods

### Data collection

The records of patients diagnosed with paragonimiasis between January 2011 and May 2019 in the Department of Infectious Diseases, Children's Hospital of Fudan University were retrospectively collected.

### Patient evaluation and diagnosis

The diagnostic criteria for paragonimiasis were based on epidemiological history: eating raw or improper cooked freshwater crab or crayfish, or drinking raw stream water; clinical manifestations: cough, chest pain, blood phlegm or pleural lesions, etc.; the elevated proportion and absolute value of peripheral eosinophil; positive anti-parasite serological test; radiographical findings; positive parasitological examination results.

### Treatment and follow-up management

A total dosage of 150 mg/kg praziquantel (PZQ) taken three times daily with meals for 3 days was recommended as a standard course. Hematological and serological examinations and imageological examinations were performed after treatment to evaluate patient recovery from the infection. Another course of PZQ or triclabendazole was used in patients with unsatisfactory responses.

### Ethics declarations

This study was conducted in accordance with the International Council for Harmonisation Guidelines for Good Clinical Practice and the Declaration of Helsinki. This study was approved by the Research Ethics Board of Children’s Hospital of Fudan University ((2020)506), with a waiver regarding informed consent.

## Data Availability

The datasets used and analyzed during the current study are available from the corresponding author on reasonable request.
