# Author Correction: A retrospective clinical analysis of pediatric paragonimiasis in a Chinese children’s hospital from 2011 to 2019

**DOI:** 10.1038/s41598-021-90897-x

**Published:** 2021-06-08

**Authors:** Manning Qian, Fei Li, Yuhan Zhang, Zhongwei Qiao, Yingyan Shi, Jun Shen

**Affiliations:** 1 Children’s Hospital of Fudan University, Shanghai, 201102 China; 2 Shanghai Medical College of Fudan University, Shanghai, 200032 China; 3 Department of Infectious Disease, Children’s Hospital of Fudan University, 399 WanYuan Road, Shanghai, 201102 China; 4 Department of Radiology, Children’s Hospital of Fudan University, 399 WanYuan Road, Shanghai, 201102 China

Correction to: *Scientific Reports* 10.1038/s41598-021-81694-7, published online 21 January 2021

In the original version of this Article, Yingyan Shi was incorrectly affiliated with ‘Department of Infectious Disease, Children’s Hospital of Fudan University, 399 WanYuan Road, Shanghai, 201102, China’. The correct affiliation is listed below.

Department of Radiology, Children's Hospital of Fudan University, 399 WanYuan Road, Shanghai, 201102, China

Additionally, Jun Shen was incorrectly affiliated with ‘Department of Radiology, Children’s Hospital of Fudan University, 399 WanYuan Road, Shanghai, 201102, China’. The correct affiliation is listed below.

Department of Infectious Disease, Children’s Hospital of Fudan University, 399 WanYuan Road, Shanghai, 201102, China

These errors have now been corrected in the PDF and HTML versions of the Article.

